# Supplementary material for: Misshapen coordinates protrusion restriction and actomyosin contractility during collective cell migration
Source: Nat Commun. 2019 Sep 2;10:3940. doi: 10.1038/s41467-019-11963-7 (PMC6718686; doi:10.1038/s41467-019-11963-7)
Supplement: Supplementary file 1 — Supplementary_info_NEW [file 41467_2019_11963_MOESM1_ESM.pdf]

Misshapen coordinates protrusion restriction and actomyosin contractility during collective cell migration

Plutoni et al. 2019

Supplementary Figures and References

Supplementary Figures

a

Drosophila identifier	Common name (Symbol)	dsRNA line number	C.I.	M.I.	*n*	Human orthologs
Control (Ctrl)	mCherry	bl35785	0.99	0.99	209	
CG18582	mushroom bodies tiny (mbt)	v109880	0.98	1	88	PAK4, PAK5, PAK6, PAK3, PAK2, PAK1
CG7693	frayed (fray)	v106919	0.96	0.99	25	OXSR1, STK39, STRADB, STRADA
		v41718	0.95	0.98	103	
CG7097	happyhour (hppy)	v35166	0.94	0.97	100	MAP4K5, MAP4K3, MAP4K2, MAP4K1
CG4527	Sterile20-like kinase (Slik)	v35179	1	1	161	STK10, SLK
		v43783	0.96	0.98	83	
		v43784	0.88	0.93	154	
CG5125	neither inactivation nor afterpotential C (ninaC)	v27359	0.98	0.99	165	MYO3A, MYO3B, TNIK, MYO9B, MYO9A, MYO7B, MYO7A, MYO15A, MYO10, MINK1, MAP4K5, MAP4K4, MAP4K3
		v110702	0.99	1.00	116	
		v27360	0.75	0.88	378	
CG5169	Germinal centre kinase III (GckIII)	v49558	0.99	1.00	157	STK26, STK24, STK25, STK4, STK3, MAP3K8
		v107158	0.99	1.00	76	
		v22024	0.73	0.91	132	
CG11228	hippo (hpo)	bl33614	0.98	0.99	75	STK3, STK4
		bl35176	1.00	1.00	61	
		v7823	0.84	0.91	212	
		v104169	0.44	0.56	101	
		bl27661	0.8	0.89	265	
CG10295	p21-activated kinase (Pak)	v108937	0.95	0.97	209	PAK1, PAK3, PAK2, PAK6, PAK5, PAK4
		v12553	0.76	0.88	171	
CG14895	Pak3 (Pak3)	v39843	0.59	0.77	162	PAK3, PAK1, PAK2, PAK6, PAK5, PAK4
		v107260	0.53	0.73	62	
		v44607	0.52	0.68	107	
CG14217	Tao (Tao)	v107645	0	0.06	55	TAOK3, TAOK1, TAOK2
		v17432	0.13	0.19	201	
CG16973	misshapen (msn)	bl28791	0	0.07	290	MINK1, MAP4K4, TNIK, NRK, MYO3B, MYO3A
		v101517	0.01	0.01	114	

b

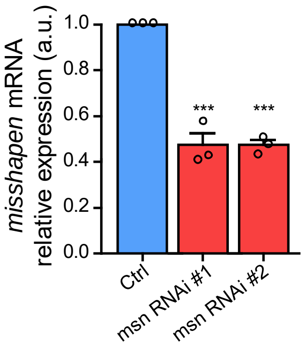

c

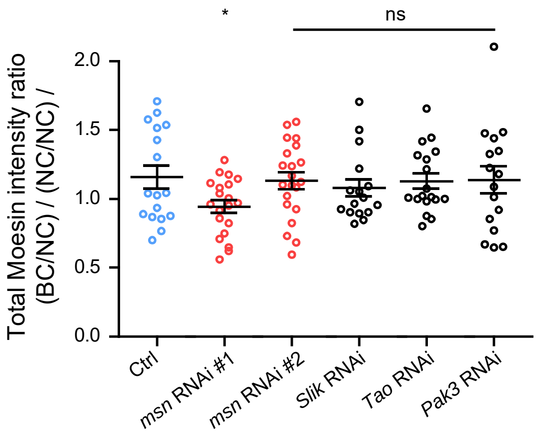

Supplementary Fig. 1: Details of the candidate RNAi screen.
a. Table representing the *Drosophila* identifiers, the common name, the dsRNA number, the completion index (C.I.), the migration index (M.I.), the number of analyzed egg chamber (n) and the Human protein orthologs corresponding to each kinase targeted. **b**. Quantification of the relative mRNA amount of *msn* in adult flies after expressing *mCherrry* RNAi (Ctrl), *msn* RNAi #1 or #2. (n = 3 independent mRNA extractions from 15 females each for each conditions) **c.** Quantification of the ratio between the mean total Moesin fluorescence intensity at the cluster periphery (BC-NC) and at the nurse cell interface (NC-NC) for the indicated RNAi lines. mCherry RNAi (n = 17), *msn* RNAi #1 (n = 20), *msn* RNAi #2 (n = 21), *Slik* RNAi (n = 16), *Tao* RNAi (n = 18) and *Pak3* RNAi (n = 15). n represents the number of independent BC cluster. Non-significant (ns) *p* > 0,05; * *p* < 0,05, one way ANOVA test coupled with Bonferroni correction methods Error bars show s.e.m.

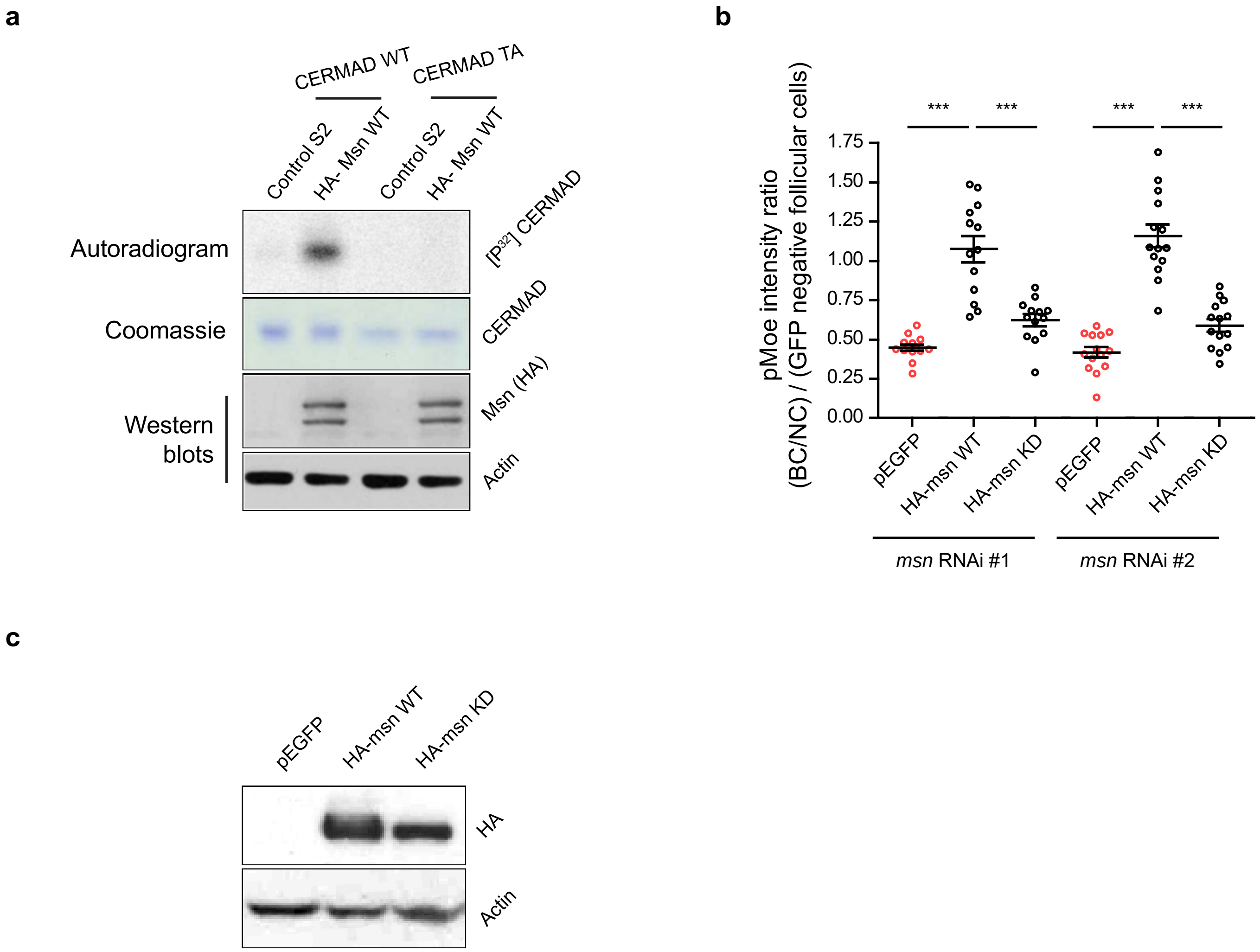

Supplementary Fig, 2. Misshapen phosphorylates the T556 residue of Moesin *in vitro* and phosphorylates Moesin *in vivo* dependently of it kinase activity.

a. Immunoprecipitated wild type HA-tagged Msn was used in kinase reactions on the CERMAD WT or CERMAD T556A domain of Moesin. Reactions were analyzed by autoradiography, western blots, and Coomassie. **b**. Quantification of the ratio of pMoe mean fluorescence intensity at the cluster periphery, normalized to the signal between nurse cells, in Controls (n= 14), *msn* RNAi#1 (n = 13), *msn* RNAi#2 (n = 13), *Slik* RNAi (n = 15), *Tao* RNAi (n = 14) and *Pak3* RNAi (n = 14). n represents the number of independent BC clusters. **c**. Western blot from Drosophila extract showing the level of expression of ubiquitously expressed HA-tagged Msn WT or Msn^{D160N}. Expression was driven by the Heat Shock GAL4 driver. *** p < 0,001, one way ANOVA test coupled with Bonferroni correction methods Error bars show s.e.m.

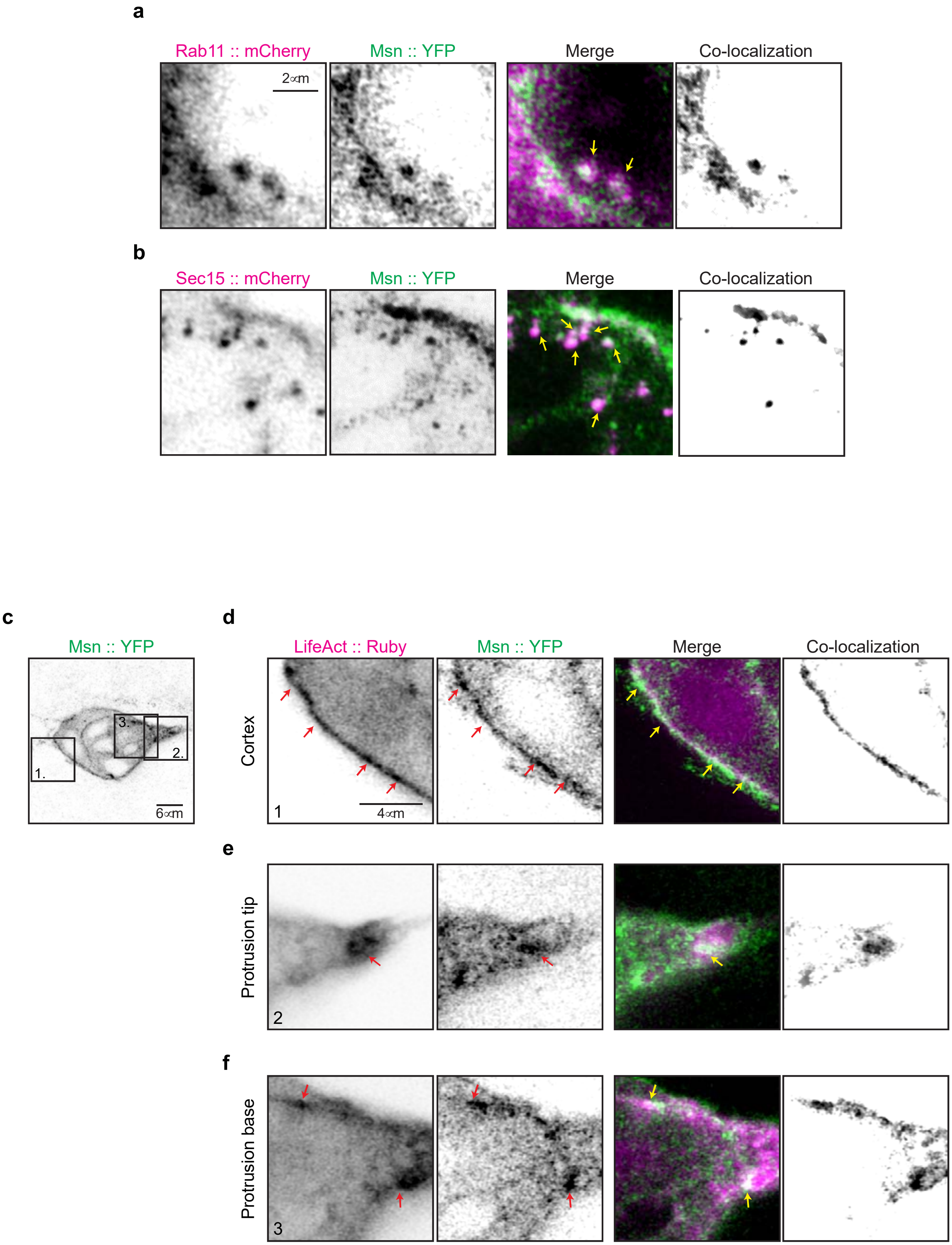

Supplementary Fig. 3: Msn localizes on Rab11 and Sec15 vesicules and on actin rich regions.

a-b Representative images showing the localization of Msn::YFP with Rab11::mCherry or Sec15::mCherry on vesicles. Images are shown as separated channels, merged images and co-localization images. Yellow arrows indicate region where both proteins co-localize.

c-f. Representative images showing the localization of Msn and actin (LifeAct::GFP) at the cortex (1), the tips of the protrusion (2) and the base of protrusions (3) represented as in a-b. Yellow arrows indicate region where both proteins co-localize.

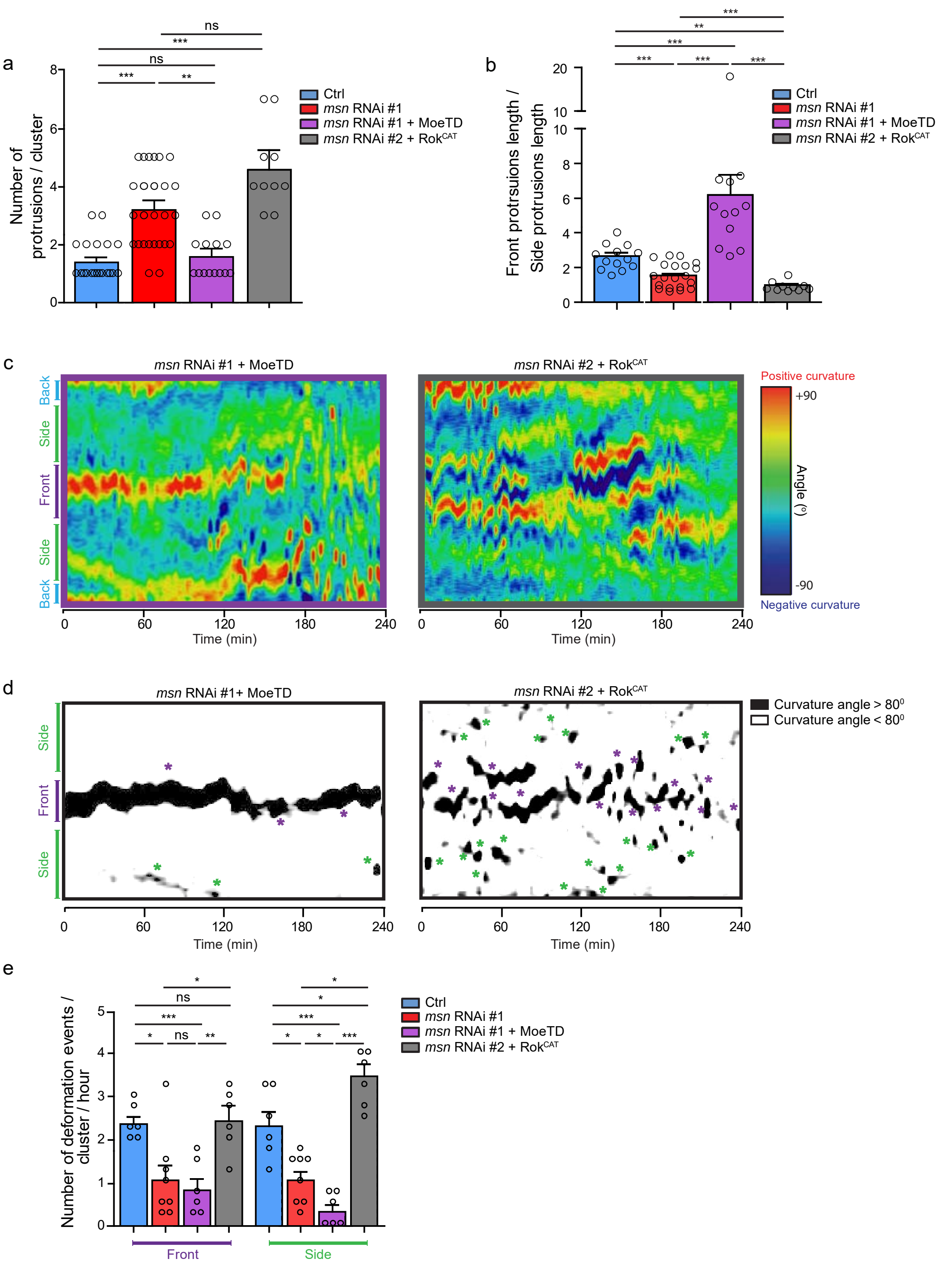

Supplementary Fig. 4: Msn acts on independent pathways to regulate protrusion restriction and contractility.

a. Number of protrusions per cluster in control conditions (n = 21), after depletion of Msn (n = 26) or in Msn-depleted clusters expressing Moe^{T556D} (n = 14) or Rok^{CAT} (n = 10). n represents the number of independent BC clusters **b.** Ratio of the length of front and side protrusions in control conditions (n= 13), after depletion of Msn (n = 20) or in Msn-depleted clusters expressing Moe^{T556D} (n = 12) or Rok^{CAT} (n = 10). n represents the number of independent BC clusters. **c**. Segmental curvatures from a time-lapse recording of an Msn-depleted cluster expressing Moe^{T556D} or Rok^{CAT}, represented as in Fig. 4b. **d.** Representation of thresholded curvature maps to highlight strong positive curvatures (>80^0) in an Msn-depleted cluster expressing Moe^{T556D} and Rok^{CAT}, represented as in Fig. 4e. **e.** Quantification of strong positive curvature events at the front and at the side of in control conditions (n = 6), after depletion of Msn (n = 8) or in Msn-depleted clusters expressing Moe^{T556D} (n = 6) or Rok^{CAT} (n = 6 . Non-significant (ns) p > 0,05; * p < 0,05; ** p < 0,01; *** p < 0,001, one way ANOVA test coupled with Bonferroni correction methods Error bars show s.e.m.

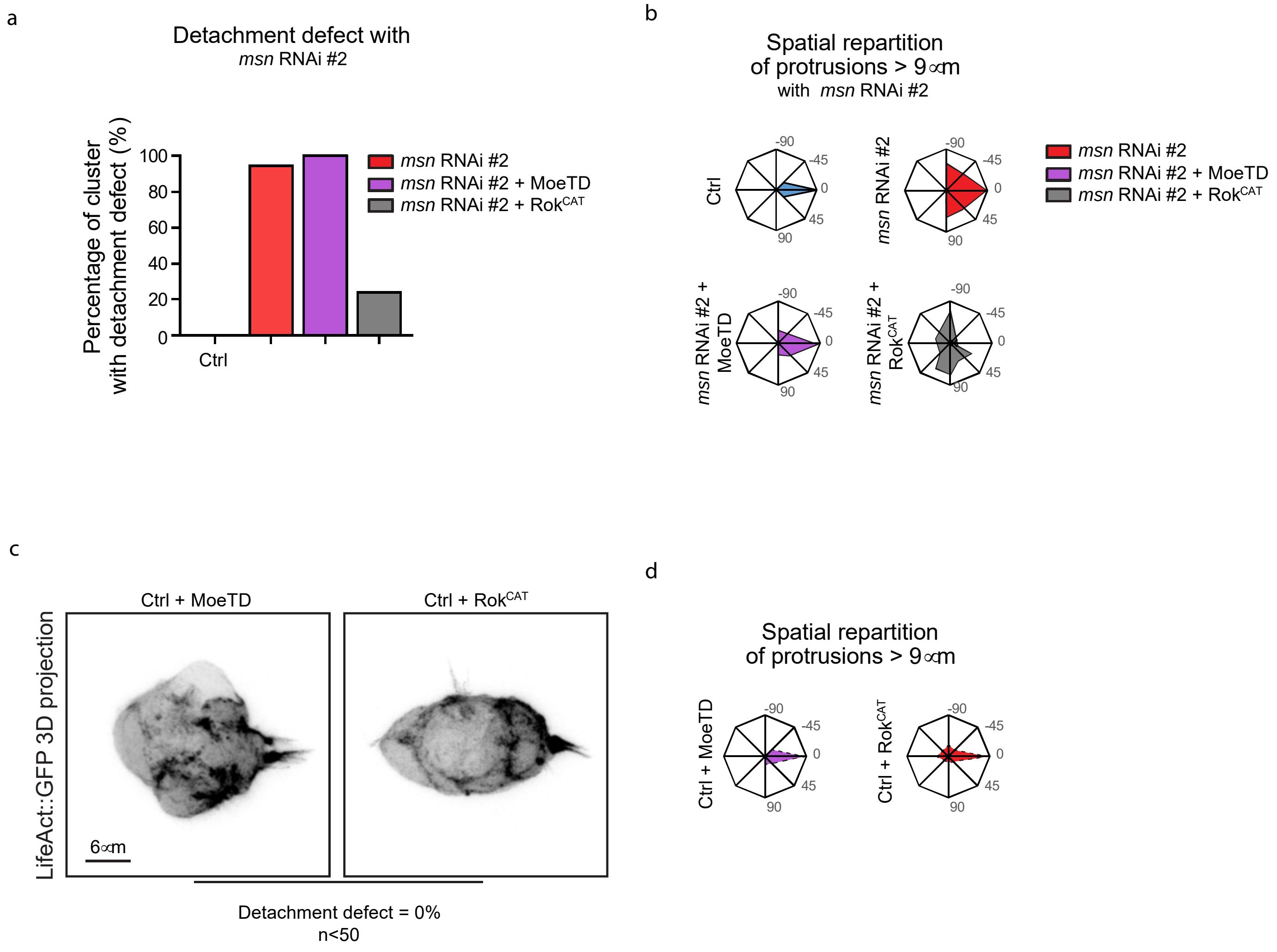

Supplementary Fig. 5: Moe^{T556D} or Rok^{CAT} **expression are not associated with detachment or protrusion defect, respectively.**

Here, we combined Moe^{T556D} or Rok^{CAT} expression with *msn* RNAi #2**. a**. Quantification of the percentage of clusters that started to migrate but have a detachment defects at stage 10 in control conditions (n = 47), after depletion of Msn (RNAi #2) (n = 18) or in Msn-depleted clusters expressing Moe^{T556D} (n = 40) or Rok^{CAT} (n = 50). (18 < n < 124).**b.** Analysis of the orientation of protrusions longer than 9 mm in control conditions (n = 20), after depletion of Msn *(msn* RNAi #2, n = 27*)* or in Msn-depleted clusters (*msn* RNAi #2*)* expressing Moe^{T556D} (n = 13) or Rok^{CAT}. (n = 10). **c.** Z-projection of a representative cluster expressing *LifeAct::GFP* and Moe^{T556D} or Rok^{CAT} in a control background. Images shown in inverted greyscale. As indicated under the images, no cluster displayed a detachment defects at stage 10 in both conditions. (n = 50). **d**. Analysis of the orientation of protrusions longer than 9 mm in control clusters that express Moe^{T556D} (n = 17) or Rok^{CAT}. (n = 16). n represents the numbers of independent BC clusters.

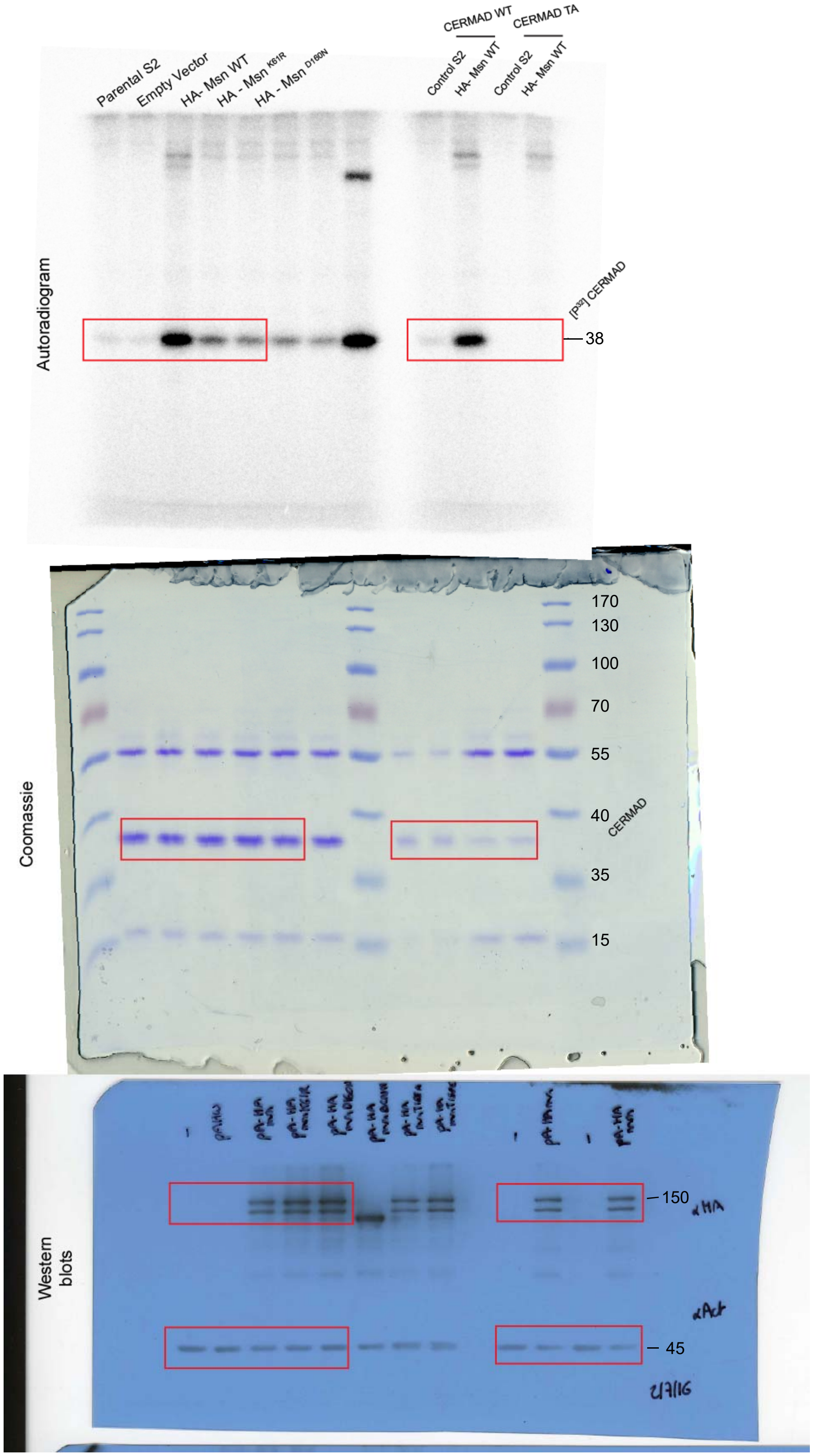

Supplementary Fig. 6

Raw autoradiograms (top panel), coomassie gel (middle panel) and western blot (bottom panel) from Fig, 1d and Supplementary Fig 2a. Immunoprecipitated wild type and kinase-dead HA-tagged Msn were used in kinase reactions on the CERMAD domain of Moesin. Reactions were analyzed by autoradiography, Coomassie and western blots. Data that are presented in the manuscript are highlighted in red.

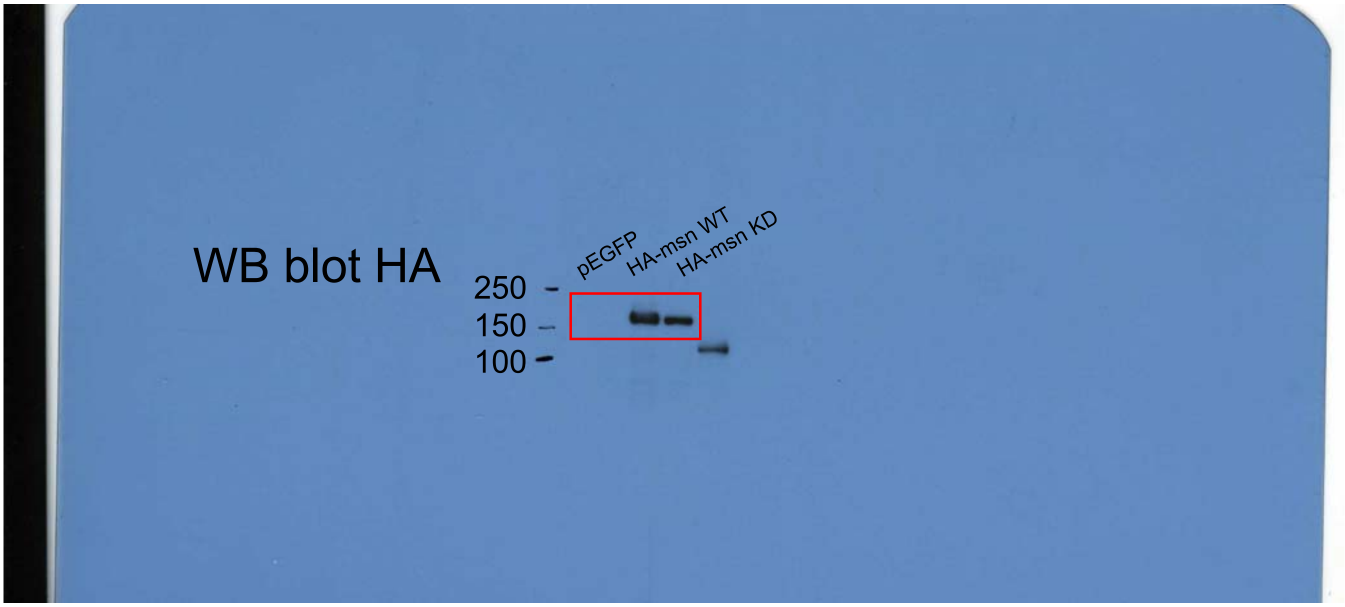

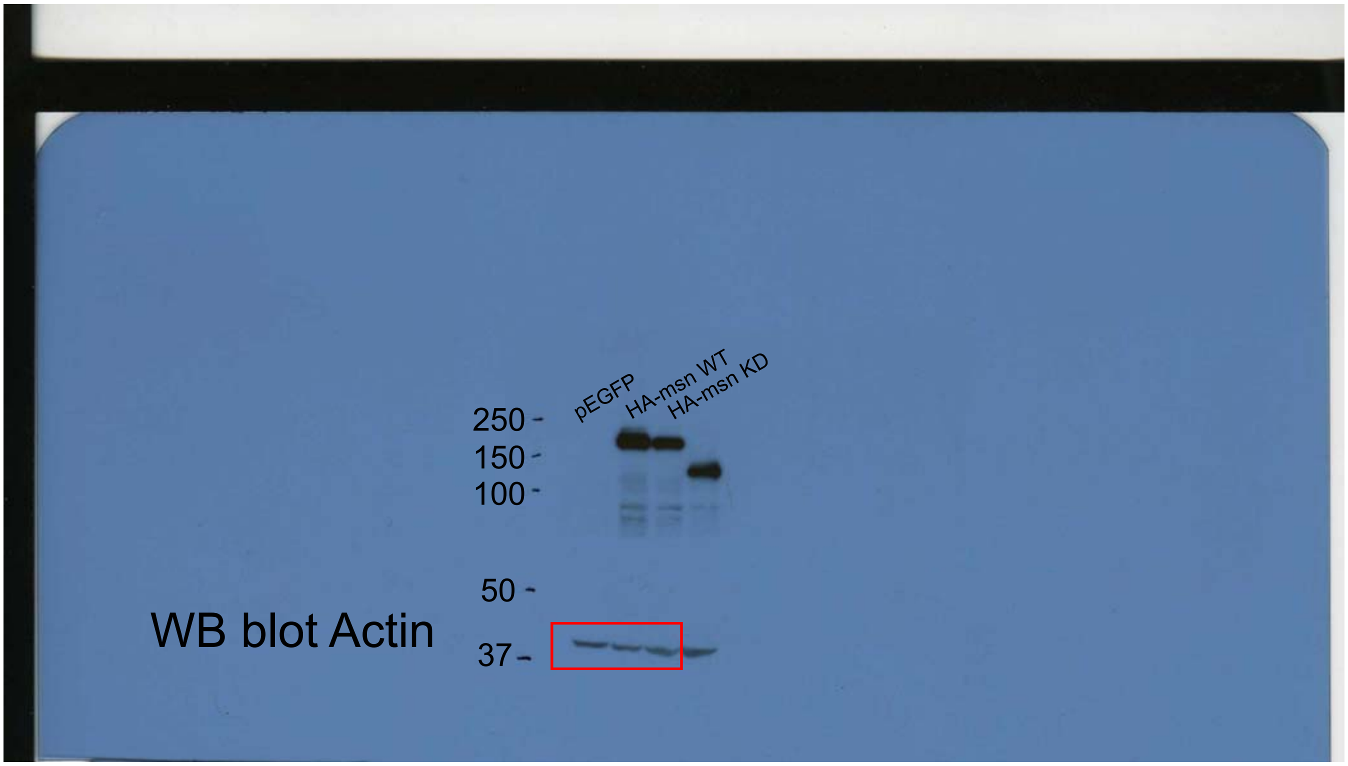

Supplementary Fig. 7

Raw western blot from Supplementary Fig. 2c. western blot from Drosophila extract showing the level of expression of ubiquitously expressed HA-tagged Msn WT or Msn^{D160N}. Expression was driven by the Heat Shock GAL4 driver. Data that are presented in the manuscript are highlighted in red.

Supplementary References

List of the fly lines used for the STE20 kinases RNAi screen in Figure. 1a and Supplementary Figure 1.a, with all the corresponding references.

Fray: v106919 [1,2] / v41718 [3-5]
Hppy: v35166 [6]
Slik: v35179 [7] / v43783 / v43784
ninaC: v27359 / v27360 [8]
GCKIII: v49558 / v107158 / v22024 [9,10]
Hippo: bl35176 [11-16] / bl35176 [14,16] / bl26661 [17] / v7823 [18] / v104169 [19-26]
Pak: v108937 [27-29] / v12553 [30]
Pak3: v39843 [28,31,32] / v107260 [31] / v44607 [28,31,32]
Tao: v107645 [22,33-35]/ v17432 [22,30,33-36]
msn: bl28791 [23,33,37] / v101517 [9,33,38,39]

1 Matzat, T. *et al.* Axonal wrapping in the Drosophila PNS is controlled by glia-derived neuregulin homolog Vein. *Development* **142**, 1336-1345, doi:10.1242/dev.116616 (2015).

2 Serysheva, E. *et al.* Wnk kinases are positive regulators of canonical Wnt/beta-catenin signalling. *EMBO Rep* **14**, 718-725, doi:10.1038/embor.2013.88 (2013).

3 Neely, G. G. *et al.* A genome-wide Drosophila screen for heat nociception identifies alpha2delta3 as an evolutionarily conserved pain gene. *Cell* **143**, 628-638, doi:10.1016/j.cell.2010.09.047 (2010).

4 Schnorrer, F. *et al.* Systematic genetic analysis of muscle morphogenesis and function in Drosophila. *Nature* **464**, 287-291, doi:10.1038/nature08799 (2010).

5 Neely, G. G. *et al.* A global in vivo Drosophila RNAi screen identifies NOT3 as a conserved regulator of heart function. *Cell* **141**, 142-153, doi:10.1016/j.cell.2010.02.023 (2010).

6 Resnik-Docampo, M. & de Celis, J. F. MAP4K3 is a component of the TORC1 signalling complex that modulates cell growth and viability in Drosophila melanogaster. *PLoS One* **6**, e14528, doi:10.1371/journal.pone.0014528 (2011).

7 Perkins, L. A. *et al.* The Transgenic RNAi Project at Harvard Medical School: Resources and Validation. *Genetics* **201**, 843-852, doi:10.1534/genetics.115.180208 (2015).

8 Petzoldt, A. G. *et al.* DE-Cadherin regulates unconventional Myosin ID and Myosin IC in Drosophila left-right asymmetry establishment. *Development* **139**, 1874-1884, doi:10.1242/dev.047589 (2012).

9 Wong, H. W., Shaukat, Z., Wang, J., Saint, R. & Gregory, S. L. JNK signaling is needed to tolerate chromosomal instability. *Cell Cycle* **13**, 622-631, doi:10.4161/cc.27484 (2014).

10 Song, Y., Eng, M. & Ghabrial, A. S. Focal defects in single-celled tubes mutant for Cerebral cavernous malformation 3, GCKIII, or NSF2. *Dev Cell* **25**, 507-519, doi:10.1016/j.devcel.2013.05.002 (2013).

11 Nagarkar-Jaiswal, S. *et al.* A library of MiMICs allows tagging of genes and reversible, spatial and temporal knockdown of proteins in Drosophila. *Elife* **4**, doi:10.7554/eLife.05338 (2015).

12 Moeller, M. E. *et al.* Warts Signaling Controls Organ and Body Growth through Regulation of Ecdysone. *Curr Biol* **27**, 1652-1659 e1654, doi:10.1016/j.cub.2017.04.048 (2017).

13 Li, D. *et al.* miR-285-Yki/Mask double-negative feedback loop mediates blood-brain barrier integrity in Drosophila. *Proc Natl Acad Sci U S A* **114**, E2365-E2374, doi:10.1073/pnas.1613233114 (2017).

14 Sopko, R. *et al.* Combining genetic perturbations and proteomics to examine kinase-phosphatase networks in Drosophila embryos. *Dev Cell* **31**, 114-127, doi:10.1016/j.devcel.2014.07.027 (2014).

15 Kwon, Y. *et al.* The Hippo signaling pathway interactome. *Science* **342**, 737-740, doi:10.1126/science.1243971 (2013).

16 Ni, J. Q. *et al.* A genome-scale shRNA resource for transgenic RNAi in Drosophila. *Nat Methods* **8**, 405-407, doi:10.1038/nmeth.1592 (2011).
17 Genevet, A. *et al.* The Hippo pathway regulates apical-domain size independently of its growth-control function. *J Cell Sci* **122**, 2360-2370, doi:10.1242/jcs.041806 (2009).
18 Liu, B. *et al.* Toll Receptor-Mediated Hippo Signaling Controls Innate Immunity in Drosophila. *Cell* **164**, 406-419, doi:10.1016/j.cell.2015.12.029 (2016).
19 Mach, J. *et al.* Modulation of the Hippo pathway and organ growth by RNA processing proteins. *Proc Natl Acad Sci U S A* **115**, 10684-10689, doi:10.1073/pnas.1807325115 (2018).
20 Hevia, C. F., Lopez-Varea, A., Esteban, N. & de Celis, J. F. A Search for Genes Mediating the Growth-Promoting Function of TGFbeta in the Drosophila melanogaster Wing Disc. *Genetics* **206**, 231-249, doi:10.1534/genetics.116.197228 (2017).
21 Su, T., Ludwig, M. Z., Xu, J. & Fehon, R. G. Kibra and Merlin Activate the Hippo Pathway Spatially Distinct from and Independent of Expanded. *Dev Cell* **40**, 478-490 e473, doi:10.1016/j.devcel.2017.02.004 (2017).
22 Poon, C. L., Mitchell, K. A., Kondo, S., Cheng, L. Y. & Harvey, K. F. The Hippo Pathway Regulates Neuroblasts and Brain Size in Drosophila melanogaster. *Curr Biol* **26**, 1034-1042, doi:10.1016/j.cub.2016.02.009 (2016).
23 Nie, Y. *et al.* Bunched and Madm Function Downstream of Tuberous Sclerosis Complex to Regulate the Growth of Intestinal Stem Cells in Drosophila. *Stem Cell Rev* **11**, 813-825, doi:10.1007/s12015-015-9617-5 (2015).
24 Brittle, A., Thomas, C. & Strutt, D. Planar polarity specification through asymmetric subcellular localization of Fat and Dachsous. *Curr Biol* **22**, 907-914, doi:10.1016/j.cub.2012.03.053 (2012).
25 Rauskolb, C., Pan, G., Reddy, B. V., Oh, H. & Irvine, K. D. Zyxin links fat signaling to the hippo pathway. *PLoS Biol* **9**, e1000624, doi:10.1371/journal.pbio.1000624 (2011).
26 Reddy, B. V. & Irvine, K. D. Regulation of Drosophila glial cell proliferation by Merlin-Hippo signaling. *Development* **138**, 5201-5212, doi:10.1242/dev.069385 (2011).
27 Wang, C. H. *et al.* USP5/Leon deubiquitinase confines postsynaptic growth by maintaining ubiquitin homeostasis through Ubiquilin. *Elife* **6**, doi:10.7554/eLife.26886 (2017).
28 Dent, L. G. *et al.* The GTPase regulatory proteins Pix and Git control tissue growth via the Hippo pathway. *Curr Biol* **25**, 124-130, doi:10.1016/j.cub.2014.11.041 (2015).
29 Langen, M. *et al.* Mutual inhibition among postmitotic neurons regulates robustness of brain wiring in Drosophila. *Elife* **2**, e00337, doi:10.7554/eLife.00337 (2013).
30 Neuert, H., Yuva-Aydemir, Y., Silies, M. & Klambt, C. Different modes of APC/C activation control growth and neuron-glia interaction in the developing Drosophila eye. *Development* **144**, 4673-4683, doi:10.1242/dev.152694 (2017).

31 Hattori, Y. *et al.* Sensory-neuron subtype-specific transcriptional programs controlling dendrite morphogenesis: genome-wide analysis of Abrupt and Knot/Collier. *Dev Cell* **27**, 530-544, doi:10.1016/j.devcel.2013.10.024 (2013).
32 Ozdowski, E. F., Gayle, S., Bao, H., Zhang, B. & Sherwood, N. T. Loss of Drosophila melanogaster p21-activated kinase 3 suppresses defects in synapse structure and function caused by spastin mutations. *Genetics* **189**, 123-135, doi:10.1534/genetics.111.130831 (2011).
33 Li, Q. *et al.* Ingestion of Food Particles Regulates the Mechanosensing Misshapen-Yorkie Pathway in Drosophila Intestinal Growth. *Dev Cell* **45**, 433-449 e436, doi:10.1016/j.devcel.2018.04.014 (2018).
34 Poon, C. L., Lin, J. I., Zhang, X. & Harvey, K. F. The sterile 20-like kinase Tao-1 controls tissue growth by regulating the Salvador-Warts-Hippo pathway. *Dev Cell* **21**, 896-906, doi:10.1016/j.devcel.2011.09.012 (2011).
35 Boggiano, J. C., Vanderzalm, P. J. & Fehon, R. G. Tao-1 phosphorylates Hippo/MST kinases to regulate the Hippo-Salvador-Warts tumor suppressor pathway. *Dev Cell* **21**, 888-895, doi:10.1016/j.devcel.2011.08.028 (2011).
36 Huang, X. *et al.* The sterile 20-like kinase tao controls tissue homeostasis by regulating the hippo pathway in Drosophila adult midgut. *J Genet Genomics* **41**, 429-438, doi:10.1016/j.jgg.2014.05.007 (2014).
37 Ugrankar, R. *et al.* Drosophila glucome screening identifies Ck1alpha as a regulator of mammalian glucose metabolism. *Nat Commun* **6**, 7102, doi:10.1038/ncomms8102 (2015).
38 Ghezzi, A. *et al.* Alcohol-induced histone acetylation reveals a gene network involved in alcohol tolerance. *PLoS Genet* **9**, e1003986, doi:10.1371/journal.pgen.1003986 (2013).
39 Marchal, C. *et al.* The HIV-1 Vpu protein induces apoptosis in Drosophila via activation of JNK signaling. *PLoS One* **7**, e34310, doi:10.1371/journal.pone.0034310 (2012).
