# Supplementary material for: Misshapen coordinates protrusion restriction and actomyosin contractility during collective cell migration
Source: Nat Commun. 2019 Sep 2;10:3940. doi: 10.1038/s41467-019-11963-7 (PMC6718686; doi:10.1038/s41467-019-11963-7)
Supplement: Supplementary file 3 — Description of Additional Supplementary Files [file 41467_2019_11963_MOESM3_ESM.pdf]

Description of Additional Supplementary Files

File name: Supplementary Movie 1
Description: Msn is necessary for BC migration. Automatic tracking of the migration of a control cluster (top) or an Msn-depleted cluster (bottom), both expressing *LifeAct::GFP*. Time-lapse recording was performed on a spinning disk confocal. A circle and a red line outline the cluster and its movement, respectively. Time interval: 3 minutes.

File name: Supplementary Movie 2
Description: Msn kinase is necessary for BC detachment and protrusion restriction Spinning disk time-lapse recording of the migration of a control cluster (top) or an Msn-depleted cluster (bottom), both expressing *LifeAct::GFP* shown in inverted greyscale. This movie outlines ectopic protrusion formation and the detachment defect observed in Msn-depleted clusters. Black arrows point to protrusions and the red arrow points to the detachment defects in Msn-depleted cluster. Time interval: 3 minutes.

File name: Supplementary Movie 3
Description: 3D representation of a BC cluster presenting a single large protrusion at the front and smaller protrusions on the side. 3D representation of a control cluster expressing *LifeAct::GFP* (green) and stained for F-actin (red). The large front protrusions are outlined by a grey surface, and the small side protrusions with purple surfaces. A z-projection of this cluster is shown in Fig. 3a.

File name: Supplementary Movie 4
Description: Msn-depleted BCs present protrusion restriction and size defects.
3D representation of an Msn-depleted cluster expressing *LifeAct::GFP* (green) and stained for F-actin (red). Three large protrusions are outlined in grey surface. A z-projection of this cluster is shown in Fig. 3a.

File name: Supplementary Movie 5
Description: Active Moe rescues protrusion restriction but not the detachment defect induced by Msn-depletion. Spinning disk time-lapse recording of a cluster depleted for Msn and expressing Moe^{T556D} and *LifeAct::GFP* (shown in inverted greyscale). Black arrows points to protrusions and the red arrow to the detachment defect. Time interval 3 minutes.

File name: Supplementary Movie 6
Description: Rok^{CAT} rescues the Msn depletion-induced detachment defect but not the protrusion restriction. Spinning disk time-lapse recording of clusters depleted for Msn and expressing Rok^{CAT} and *LifeAct::GFP* (shown in inverted greyscale). Black arrows point to protrusions. Time interval 3 minutes.
